# Hospital Appointments

**Published:** 1886-04

**Authors:** 


HOSPITAL APPOINTMENTS.—The following appointments have been made at the different hospitals in this city:

At Cook County Hospital—Internes: S. C. Plummer, R. Bernauer, W. N. Hibbard, W. S. Pickard, L. J. Mitchell, E. M. Smith, G. W. Post, F. L. Marion. Externes: R. Chandler, L. Goldstein, A. J. Ochsner, W. C. Abaly.

St. Luke's Hospital—Internes: C. H. Easter, E. J. Hurie.

Presbyterian Hospital—Internes: A. J. Ochsner, G Marion.

Mercy Hospital—Internes: W. H. Marble, A. C. Broell.

